# Assessing ADHD prevalence and comorbidities in the United States: Insights from the Substance Abuse and Mental Health Services (SAMHSA) data

**DOI:** 10.1017/gmh.2024.104

**Published:** 2024-12-06

**Authors:** Mahie Patil, Sanjana Konda, Latha Ganti

**Affiliations:** 1 Orlando Science School, University of Central Florida, Orlando, FL, USA; 2 The Warren Alpert Medical School of Brown University, Providence, RI, USA; 3 Orlando College of Osteopathic Medicine, Winter Garden, FL, USA

**Keywords:** Attention deficit hyperactivity disorder, Substance Abuse and Mental Health Services Administration, ADHD comorbidities, ADHD demographics, ADHD hospitalization

## Abstract

This study analyzes 2022 data from SAMHSA’s Mental Health Client-Level Data (MH-CLD) to investigate ADHD prevalence and comorbidity. The findings reveal that 10.70% of the 5,899,698 patients were diagnosed with ADHD, indicating a high demand for targeted resources. ADHD prevalence declines with age, highest in children aged 0–11, and decreases with educational attainment, emphasizing the need for early intervention. Employment challenges are significant, with the highest ADHD prevalence among those not in the labor force. Racial disparities show Black individuals have the highest ADHD rates (9.71%) and Asian individuals the lowest (5.05%). Geographic differences indicate higher prevalence in the Midwest and South. Gender disparities and marital status also influence prevalence, with males and never-married individuals showing higher rates. ADHD shows strong comorbidity with oppositional defiant disorder, pervasive developmental disorder/autism spectrum disorder and conduct disorder. Effective ADHD management requires collaborative efforts from educators, employers, healthcare providers and policymakers to create supportive environments and tailored approaches considering demographic variables, comorbid conditions and socioeconomic factors.

## Graphical abstract

**Figure:**
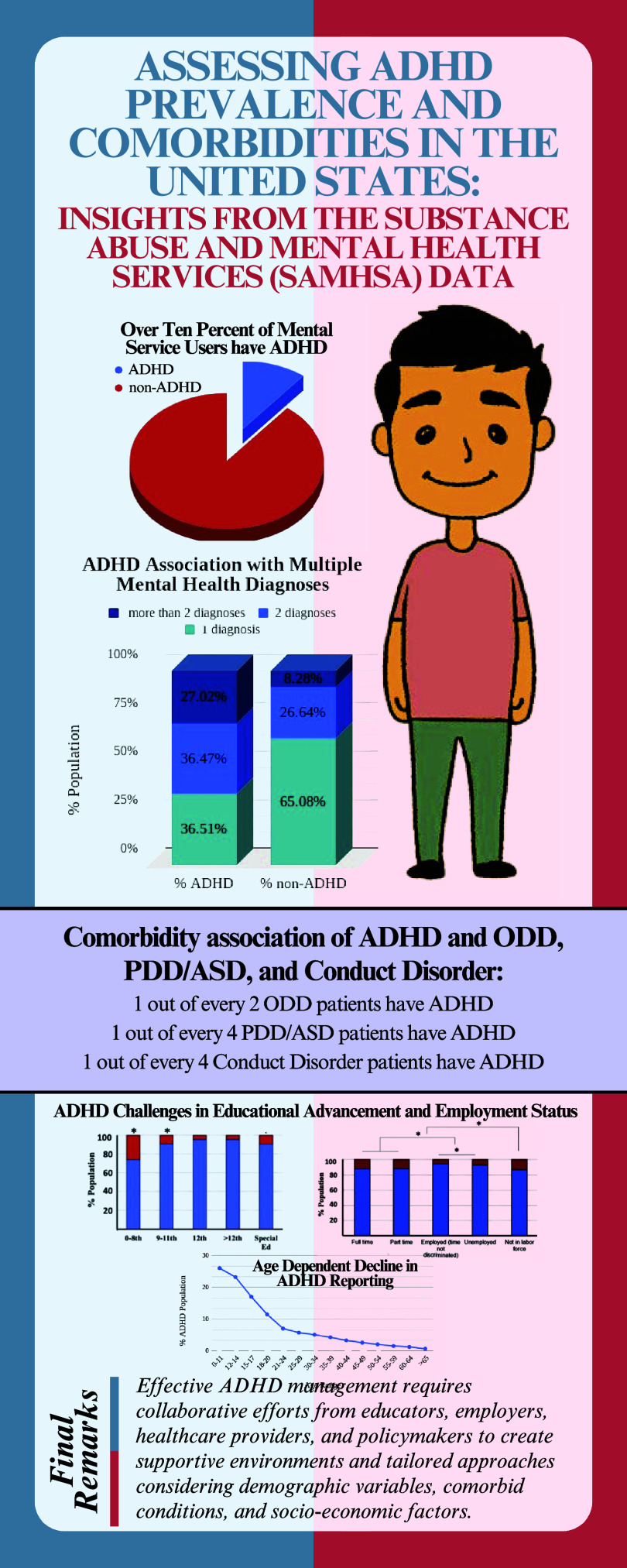

## Impact Statement

This study sheds light on the widespread impact of ADHD in the United States, revealing that over 10% of mental health service users are diagnosed with the condition. The research highlights significant differences in ADHD rates across age groups, education levels, racial backgrounds and living situations, emphasizing how social and economic factors shape its prevalence. The data shows that ADHD is more common in children and those with lower educational attainment, pointing to the need for early intervention and tailored support in schools. The data also indicates that individuals with ADHD face challenges in maintaining employment, suggesting the importance of workplace accommodations. Additionally, the variation in ADHD rates across different racial groups and regions indicates that healthcare approaches should be customized to meet the needs of diverse communities. Moreover, this analysis uncovers a strong link between ADHD and other conditions, such as oppositional defiant disorder, autism spectrum disorder and conduct disorder, suggesting the need for comprehensive healthcare approaches that address multiple conditions simultaneously. With variations in ADHD rates across racial groups and regions, the findings emphasize the importance of customizing healthcare strategies to meet the needs of diverse communities, ultimately aiming to enhance the quality of life for those affected.

## Introduction

Attention-deficit hyperactivity disorder (ADHD) is a prevalent neurodevelopmental disorder characterized by atypical levels of overactivity, inattention, and impulsivity (American Psychiatric Association, 1994). The diagnosis of ADHD primarily relies on clinical assessments based on diagnostic classification systems such as the Diagnostic and Statistical Manual of Mental Disorders, Fifth Edition (American Psychiatric Association, 2013) and the International Classification of Diseases, 11^th^ revision (World Health Organization, 2018). The DSM-5 emphasizes two core dimensions of ADHD: inattention and hyperactivity/impulsivity (American Psychiatric Association, 1994).

ADHD’s prevalence is influenced by various demographic parameters, including age, gender, socioeconomic status and geographic location. For instance, studies indicate that ADHD symptoms are more common in urban areas and in the northeastern and north-central states of the United States, suggesting environmental factors may play a role in the disorder’s manifestation (Faraone and Biederman, 2005). Additionally, the symptoms of ADHD are negatively associated with relationship quality, social life, health and education, highlighting the extensive impact of the disorder on individuals’ lives (Das et al., 2012). According to the DSM-5, an accurate diagnosis of ADHD requires that symptoms are not better explained by another mental disorder, raising important questions about the differentiation between ADHD and other conditions such as generalized anxiety disorder (GAD), substance use disorder (SUD), bipolar disorder, oppositional defiant disorder (ODD) and depression. This underscores the necessity of distinguishing mental conditions with overlapping presentations and diagnostic criteria, as well as understanding the nature of comorbid diagnoses.

Comorbidity is a significant aspect of ADHD, with many individuals diagnosed with the disorder also experiencing other psychiatric or neurodevelopmental conditions. Comorbidity, defined as the simultaneous presence of two or more conditions in a patient, is common in individuals with ADHD. ADHD is associated with a high rate of psychiatric comorbidity due to its diverse neurocognitive impairments and the wide range of related brain anomalies (Faraone et al., 2015). For instance, a large nationwide study in Denmark (14,825 patients aged 4–17 in Danish psychiatric hospitals) found that more than half of the subjects with ADHD had at least one comorbid psychiatric disorder, commonly conduct disorders, specific developmental disorders of language, learning and motor development, autism spectrum disorder (ASD) and intellectual disability (Jensen and Steinhausen, 2015).

Research on ADHD prevalence and comorbidity in Spanish preschoolers revealed that the most frequent comorbid conditions were ODD and tics, with ADHD-diagnosed preschoolers exhibiting a higher risk of behavioral problems, ASD, OCD and tics (Canals et al., 2018). Similarly, Kadesjö et al. found that 87% of Swedish school-age children with ADHD had comorbid diagnoses, predominantly ODD and developmental coordination disorder (Kadesjö and Gillberg, 2001). These findings underscore the complexity of ADHD and the importance of considering comorbid conditions in its diagnosis and treatment. A six-year follow-up study by Riddle et al. on the clinical course of ADHD symptom severity and diagnosis found that comorbidity with ODD or conduct disorder was associated with a 30% higher risk of persistent ADHD and greater hyperactivity/impulsivity (Riddle et al., 2013). The study concluded that comorbid ODD or CD during follow-up was a strong predictor of diagnostic stability, indicating that comorbidity may contribute to the degree of impairment and the long-term progression of symptoms (Riddle et al., 2013).

A systematic review and meta-analysis highlighted a 28% prevalence of comorbid ADHD in individuals with ASD, emphasizing the need for careful assessment and tailored interventions for these populations (Lai et al., 2019). The DSM-5 revisions in 2013 reflect this understanding, allowing the diagnosis of ADHD in the presence of ASD due to the high comorbidity between the two disorders (Leffa et al., 2022).

Regarding demographic information, ADHD symptoms negatively impact relationship quality, social life, health and education (Das et al., 2012). A population-based study indicated that ADHD symptoms are positively associated with financial problems and unemployment (World Health Organization, 2018). Another study by Faraone and Biederman found ADHD to be more common in urban areas and northeastern and north-central states, suggesting that individuals with ADHD may select environments that accommodate their symptoms, such as the faster pace of urban living (Faraone and Biederman, 2005). The study also indicated that individuals with narrow ADHD are 2.6 times more likely to be unemployed and less likely to complete high school, attend college or graduate (Faraone and Biederman, 2005). Understanding the demographic parameters influencing ADHD prevalence and the existence of comorbid conditions is crucial for healthcare providers. It aids in the development of targeted diagnostic criteria, treatment plans and support systems tailored to the needs of specific populations. For example, recognizing the higher prevalence of ADHD in urban areas can inform resource allocation and the creation of specialized programs in those regions. Additionally, addressing comorbid conditions can enhance the overall quality of care and improve outcomes for individuals with ADHD.

This study aims to further investigate ADHD prevalence and comorbidity using data from the Substance Abuse and Mental Health Services Administration’s (SAMHSA) Mental Health Client-Level Data (MH-CLD) collected during 2022. By exploring correlations and contingency/associations between different mental health conditions diagnosed with ADHD and analyzing demographic information, this research seeks to inform patient-specific treatment methods and contribute to the broader understanding of ADHD within the healthcare system. Ultimately, this study underscores the critical importance of addressing ADHD comprehensively to support affected individuals and optimize healthcare resources.

## Materials and methods

### Data source

The data for this study were derived from the MH-CLD Public Use File (PUF) for the year 2022, prepared for the SAMHSA under Contract No. 75S20320C00001 with SAMHSA, U.S. Department of Health and Human Services (HHS). This dataset provides demographic and mental health characteristics for clients who have utilized mental health services in facilities that report to individual state administrative data systems. The data are in the public domain and can be accessed from SAMHSA’s official website.

### Data collection

The MH-CLD data collection framework involves compiling demographic, clinical and outcome data for individuals served by state mental health agencies (SMHAs) over a state-defined 12-month reporting period. States have the option to use either the calendar year or the state fiscal year as their reporting period. The dataset includes individuals who received mental health and support services, including screening, assessment, crisis services and telemedicine/telehealth, from programs operated or funded by SMHAs during the reporting period. To protect confidentiality, potentially identifying variables were top- or bottom-coded. This method maintains the analytical integrity of the dataset while protecting individual privacy. The MH-CLD dataset includes clients from providers receiving public funding, but due to the variability in state funding mechanisms, the exact number of clients treated without public funding is unknown. Additionally, the dataset does not represent the total national demand for mental health treatment or the mental health status of the national population. Data limitations include incomplete reporting of mental health diagnoses, potentially leading to biased prevalence estimates. The following states and territories did not report sufficient data for the year 2022 and are excluded from the dataset: American Samoa, Federated States of Micronesia, Guam, Maine, Marshall Islands and the U.S. Virgin Islands.

### Data analysis and presentation

Data analysis was conducted using multiple software tools to ensure robust and comprehensive results. JMP Pro 15 software was utilized for initial data exploration, focusing on analyzing contingency tables and organizing groups based on various patient variables. This allowed for an in-depth understanding of the relationships and patterns within the dataset, setting the stage for more detailed statistical analysis. For most of the statistical analyses, including analysis of variance (ANOVA), pairwise comparison testing and contingency tests, Origin Pro 2024 software was used. This software’s advanced statistical tools and features allow for precise and accurate analyses. ANOVA was used to determine the statistical significance of differences between group means, while pairwise comparison testing helped in identifying specific group differences. Contingency tests were applied to examine the association between categorical variables, providing insights into the relationships within the data. Microsoft Excel was employed to generate graphs and tables, providing a clear and visually appealing presentation of the data. This facilitated easier interpretation and comparison of key findings, aiding in the communication of complex statistical results.

## Results

**Figure 1.**
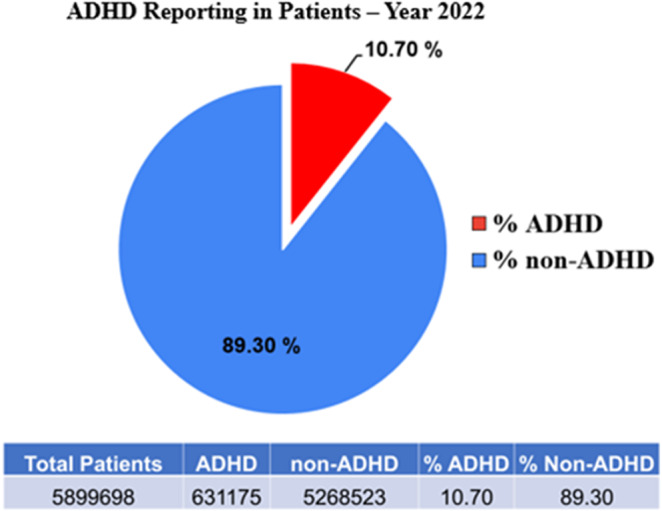
The figure illustrates the reporting of ADHD among patients in the year 2022, using a pie chart to depict the proportion of patients diagnosed with ADHD compared to those without the diagnosis. The red segment highlighting the percentage of ADHD patients and the blue segment representing non-ADHD patients. *n* = 5,899,698.

In 2022, one in ten patients reported ADHD. To understand the prevalence of ADHD in 2022, the collected patient data was analyzed. The data reveals that out of a total of 5,899,698 patients, 631,175 individuals were diagnosed with ADHD, representing 10.70% of the total patient population, underscoring the significant prevalence of ADHD within the patient population. Conversely, 5,268,523 patients, or 89.30%, were not diagnosed with ADHD (Figure 1). The data’s implications are substantial for healthcare, suggesting a notable demand for resources and support systems dedicated to ADHD diagnosis and treatment. The prevalence rate of more than one in ten patients emphasizes the need for targeted interventions and informed public health policies.

### ADHD reporting shows age-dependent decline and varies by education level

**Figure 2.**
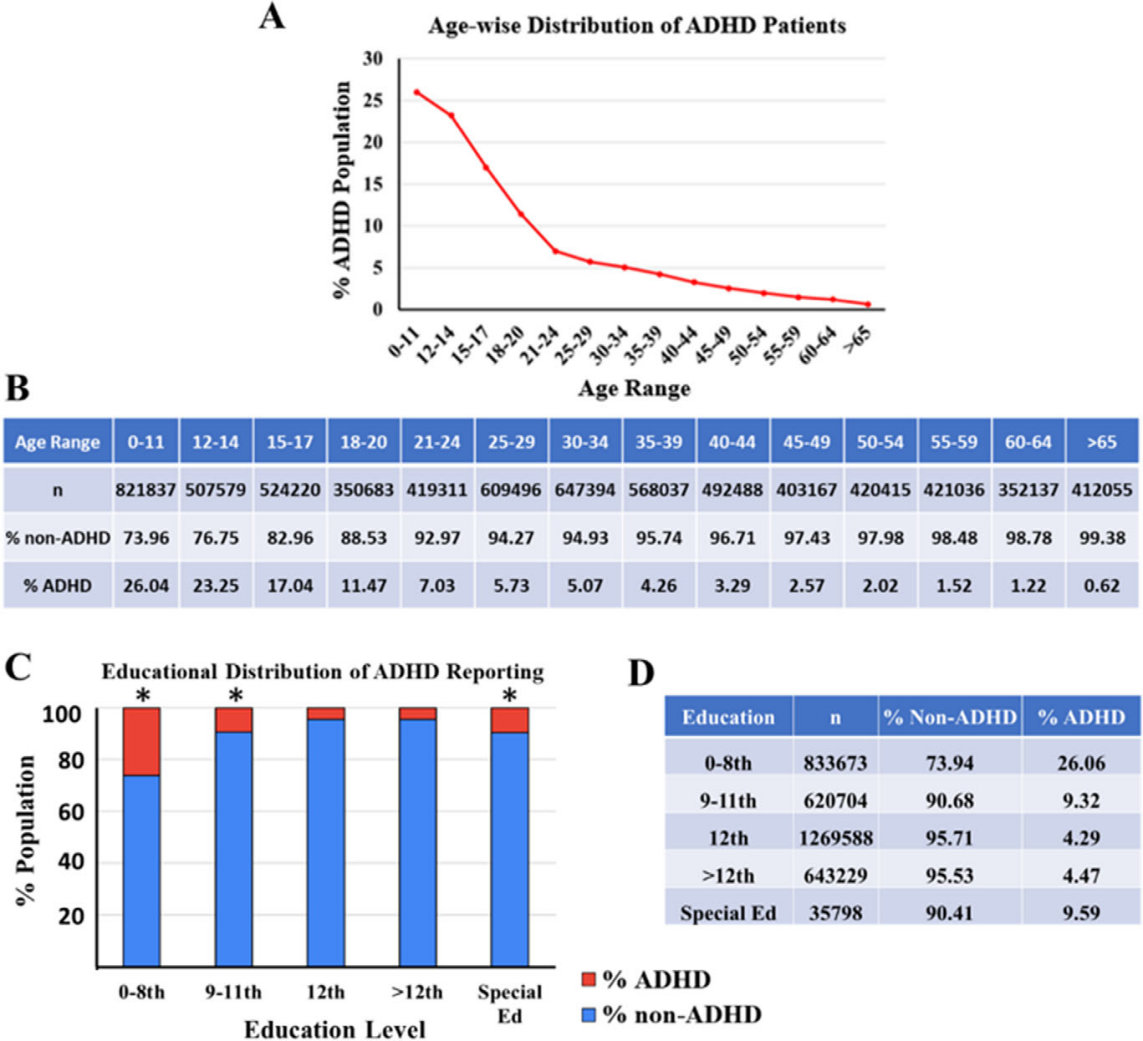
The figure presents a comprehensive analysis of the distribution of ADHD patients based on age and education level. (a) The line graph represents the decreasing prevalence of reported ADHD (% population) with respect to the various age ranges (0–11, 12–14, 15–17, 18–20, 21–24, 25–29, 30–34, 35–39, 40–44, 45–49, 50–59, 60–64 and >65 years old) in a given population from data collected in the year 2022. (b) The table represents the ADHD and non-ADHD % populations with respect to age range, along with the total number of subjects (*n*) for each age range analyzed in this study. (c) The stacked bar chart represents the prevalence % of ADHD and non-ADHD reported population with respect to current grade level/highest education completed (special education, 0-8th, 9 to 11th, 12th and above 12th grade) in the year 2022. The ADHD population is represented in red, and the non- ADHD is represented in blue. *Indicates a statistically significant difference between age groups (ANOVA followed by pairwise comparison Bonferroni test, *p* < 0.0001). (d) The accompanying table provides numerical details, including the number of individuals (*n*) in each education category and the corresponding percentages of non-ADHD and ADHD individuals.

The data collected in 2022 shows that ADHD reporting decreases with an increase of age. Ages 0–11 years have significantly higher reported cases (26.04%), followed by subsequent significant decreases in the age range 12–14 (23.25%), 15–17 (17.04%), 18–20 (11.47%) and 21–24 (7.03%) years, respectively (ANOVA followed by pairwise Bonferroni test, *p* < 0.0001). Further data analysis also shows a statistically significant linear negative correlation of ADHD reporting with age (Figure 2a,b, Pearson correlation analysis, *R*^2^ = −0.72375, *p* < 0.0001). This observation is also in alignment with the educational distribution of reported ADHD cases. The data shows a significantly higher ADHD reporting in 0–8^th^ grades, which decreases in 9–11^th^ grades, followed by another significant decrease in Special Ed (Figure 2c,d, ANOVA followed by pairwise Bonferroni test, *p* < 0.0001). Additionally, there is no significant difference between 12^th^ grade and 12+ education. These findings suggest that ADHD prevalence is inversely related to education level, with higher ADHD rates observed in individuals with lower education attainment and those in special education. This detailed analysis underscores the importance of considering both age and educational background in understanding the distribution and impact of ADHD within the population.

### ADHD has an impact on employment status

**Figure 3.**
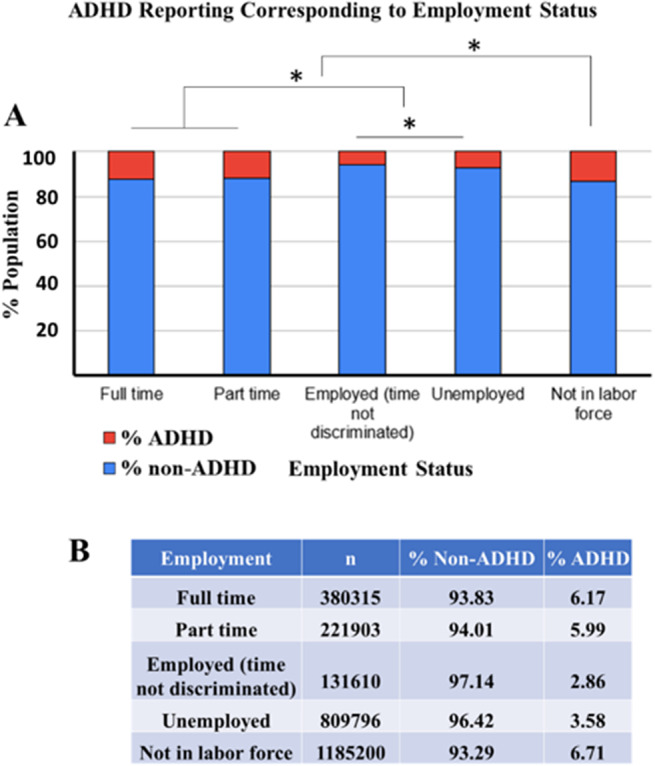
(a) The stacked bar chart represents the % ADHD and non-ADHD reported populations with respect to employment status (full-time, part-time, employed without discrimination of time, unemployed, and not in the labor force) in a given population from data collected in 2022. The ADHD population is represented in red, and the non-ADHD population is represented in blue. *Indicates a statistically true difference between groups (ANOVA followed by pairwise comparison Bonferroni test, *p* < 0.0001). (b) The table represents the ADHD and non-ADHD % populations with respect to employment status. *n* represents the total number of subjects in each category.

For a comparative analysis, various employment statuses between individuals with and without ADHD were compared. As shown in Figure 3, the most significantly high prevalence of % ADHD (6.71%) was reported among the population that is not currently in the labor force. The % ADHD prevalence among full-time and part-time employees was not different from each other but was significantly less than the population that absolutely has no income since they are not currently in the labor force. Furthermore, the % ADHD population among unemployed but in the labor force (3.58%) was significantly higher than that employed with time not discriminated (2.86%) population (Figure 3, ANOVA followed by pairwise comparison Bonferroni test, *p* < 0.0001). These findings highlight the challenges faced by individuals with ADHD in securing and maintaining employment, underscoring the need for targeted interventions to support their workforce participation.

### ADHD prevalence varies across racial groups

**Figure 4.**
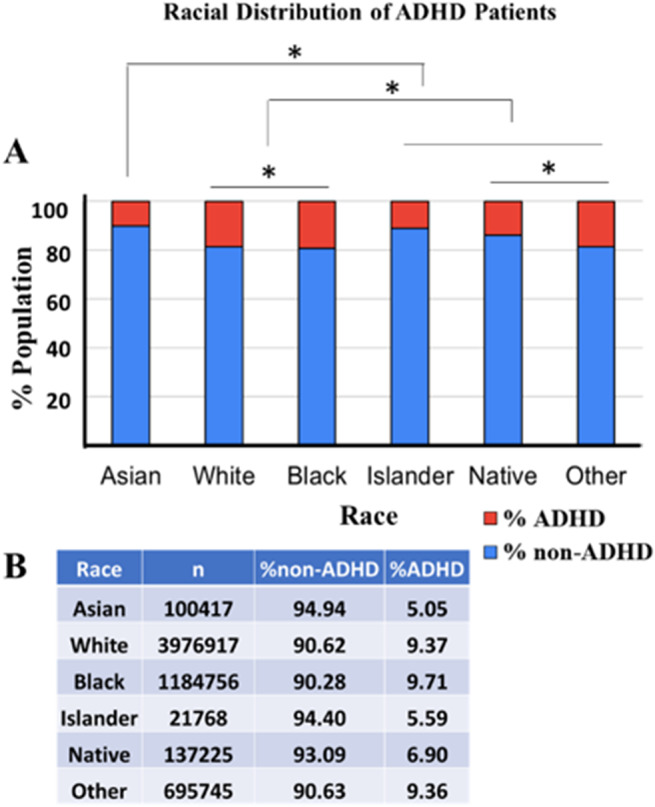
The figure provides an analysis of ADHD prevalence across different racial groups represented by a bar chart and an accompanying table. (a) The bar chart visually displays the percentage of ADHD (in red) and non-ADHD (in blue) individuals within each racial category (Asian, White, Black, Islander, Native, and Other). *Indicates a statistically significant difference between groups (ANOVA followed by pairwise comparison Bonferroni test, *p* < 0.0001). (b) The table details the number of individuals (*n*) within each racial group and the corresponding percentages of non-ADHD and ADHD patients.

From the data, it is evident that the prevalence of ADHD varies among different racial groups (Figure 4, ANOVA followed by pairwise comparison Bonferroni test, *p* < 0.0001). The Asian group has the lowest percentage of ADHD at 5.05%, with 94.94% of individuals not diagnosed with ADHD out of 100,417 total individuals. The White group, consisting of 3,976,917 individuals, shows a higher ADHD prevalence at 9.37%, with 90.62% non-ADHD individuals. The Black group has 1,184,756 individuals, with 9.71% diagnosed with ADHD and 90.28% without ADHD. Islanders have a slightly higher ADHD percentage at 5.59%, with 94.40% non-ADHD among 21,768 individuals. The Native group, with 137,225 individuals, has a 6.90% ADHD prevalence and 93.09% non-ADHD. The ‘Other’ category includes 695,745 individuals, with 9.36% ADHD and 90.63% non-ADHD. These results highlight that while ADHD is present across all racial groups, the prevalence rates vary, with Asian individuals showing the lowest and Black individuals having the highest percentages of ADHD. The data underscores the importance of considering racial differences in the prevalence and reporting of ADHD for more targeted and effective healthcare strategies.

### ADHD prevalence varies by U.S. regions and divisions

**Figure 5.**
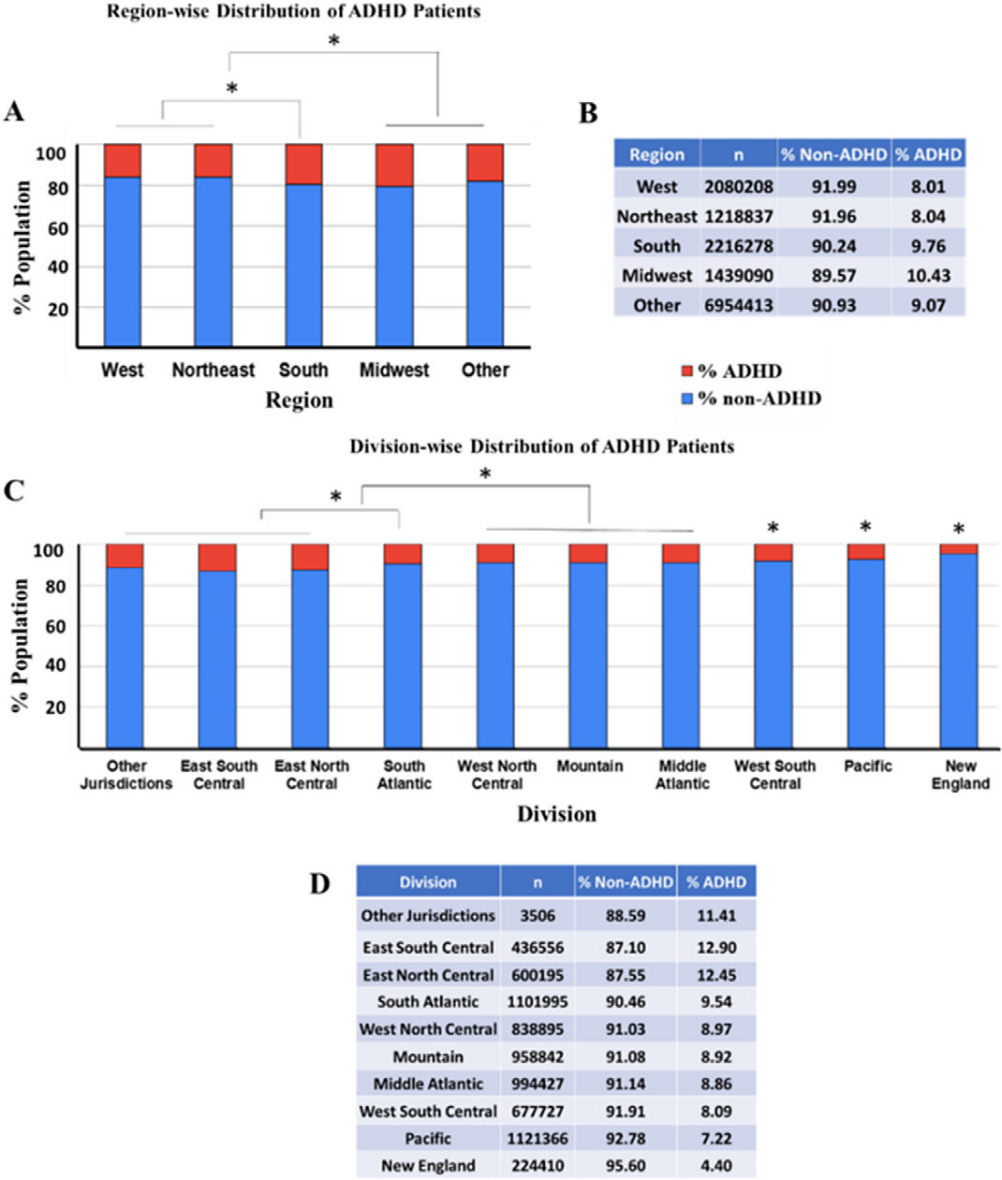
The figure provides a detailed analysis of ADHD prevalence across different U.S. regions and divisions. The bar charts and accompanying tables depict the percentage of ADHD (in red) and non-ADHD (in blue) individuals within each geographic category. *Indicates a statistically significant difference between groups (ANOVA followed by pairwise comparison Bonferroni test, *p* < 0.0001). (a) The stacked bar chart visually displays the percentage of ADHD prevalence within each regional category (West, Northeast, South, Midwest and Other). (b) The table next to the chart details the number of individuals (*n*) within each regional group and the corresponding percentages of non-ADHD and ADHD patients. (c) The stacked bar chart represents the % ADHD and non-ADHD reported populations with respect to various divisions in the United States from data collected in 2022. (d) The table represents the ADHD and non-ADHD % populations and the total number (*n*) of subjects in each category.

To understand ADHD prevalence and distribution differences across various regions and divisions in the United States, the patient data was analyzed considering five distinct regions and ten divisions. The regional distribution data shows that the West region reported an ADHD prevalence of 8.01% among 2,080,208 individuals, the Northeast at 8.04% among 1,218,837 individuals, the South at 9.76% among 2,216,278 individuals, the Midwest at 10.43% among 1,439,090 individuals and the ‘Other’ category at 9.07% among 6,954,413 individuals. The data indicates a higher ADHD prevalence reported in the Midwest, South and other regions compared to the West and Northeast (Figure 5a,b, ANOVA followed by pairwise comparison Bonferroni test, *p* < 0.0001). Prevalence in the Midwest and other regions had no significant difference.

The division-level data distribution reveals more granular differences. The divisions East South-Central division (12.90% ADHD, among 436,556 individuals), East North Central division (12.45% ADHD among 600,195 individuals) and other jurisdiction divisions (11.41% ADHD among 3506 individuals) showed significant difference between the groups and reported highest prevalence as compared to the rest of the divisions (Figure 5c,d, ANOVA followed by pairwise comparison Bonferroni test, *p* < 0.0001). Other notable divisions that followed the highest prevalence grouping include the South Atlantic division, with 9.54% ADHD prevalence among 1,101,995 individuals. The Middle Atlantic (8.86% ADHD among 994,427 individuals), West North Central (8.97% ADHD among 838,895 individuals) and Mountain (8.92% ADHD among 958,892 individuals) divisions followed the subsequent higher prevalence rates in comparison. Next, West South Central and Pacific divisions reported the comparative prevalence. The New England division reports the lowest ADHD prevalence at 4.40% among 224,410 individuals. These findings highlight significant geographic variability in ADHD prevalence, suggesting that regional and divisional factors may influence the reporting and diagnosis of ADHD. This comprehensive geographic analysis underscores the need for region-specific strategies and resources to manage ADHD across the United States effectively.

### ADHD prevalence varies significantly by sex, marital status and living arrangements

**Figure 6.**
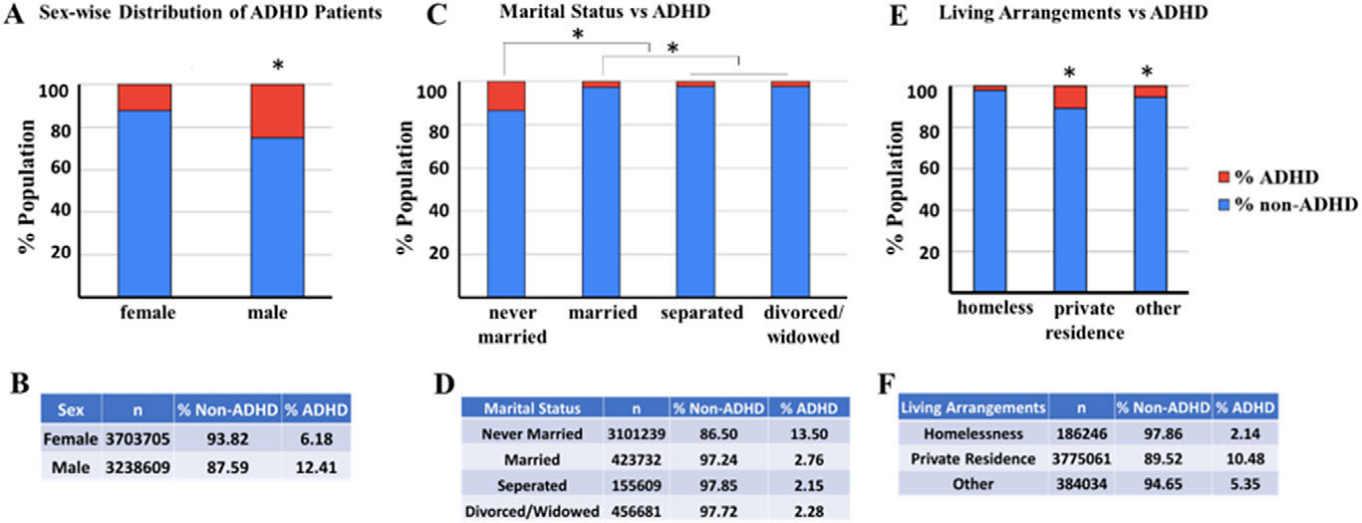
The figure provides a comprehensive analysis of ADHD prevalence across different demographics, including sex, marital status and living arrangements. The stacked bar charts and accompanying tables depict the percentage of ADHD (in red) and non-ADHD (in blue) individuals within each category. *Indicates a statistically true difference between groups (*p* < 0.0001). (a) The stacked bar chart visually displays the percentage of ADHD prevalence by sex (a), marital status (c) and living arrangements (e). The tables [(b)-sex, (d)-marital status, (f)-living arrangement] provide the number of individuals (*n*) within each demographic category and the corresponding percentages of non-ADHD and ADHD patients.

The collected data was analyzed to understand ADHD prevalence differences by gender (male, female), marital status (never married, now married, separated, divorced, widowed) and individual’s living arrangements (Experiencing homelessness, private residence, other). Figure 6 provides a comprehensive analysis of ADHD prevalence across these demographics. The ADHD prevalence analysis focused on sex reveals that males have significantly higher ADHD prevalence at 12.41% among 3,238,609 individuals compared to females at 6.18% among 3,703,705 individuals (Figure 6a,b, Pearson chi-square contingency test, *p* < 0.0001). This indicates a notable gender disparity in ADHD diagnosis, with males being more frequently diagnosed than females.

The ADHD prevalence analysis with marital status shows that individuals who have never married have the highest ADHD prevalence at 13.50% among 3,101,329 individuals (Figure 6c,d,, ANOVA followed by pairwise comparison Bonferroni test, *p* < 0.0001). In contrast, those who are married have a significantly lower prevalence at 2.76% among 4,237,732 individuals. Separated individuals have a prevalence of 2.15% among 155,609 individuals, and divorced or widowed individuals have a prevalence of 2.28% among 456,681 individuals. There is no statistical difference between separated and divorced/widowed groups. This data suggests that marital status may influence the likelihood of an ADHD diagnosis, with never-married individuals showing the highest prevalence.

The ADHD prevalence analysis with living arrangements indicates that individuals living in private residences have a significantly higher ADHD prevalence reported of 10.48% among 3,775,061 individuals (Figure 6e,f, ANOVA followed by pairwise comparison Bonferroni test, *p* < 0.0001). Those experiencing homelessness have a much lower prevalence at 2.14% among 186,246 individuals, while individuals in other living arrangements have a prevalence of 5.35% among 384,034 individuals. This suggests that living conditions might impact ADHD diagnosis rates, with private residence dwellers having a higher prevalence compared to those in homelessness or other living situations.

### Distribution patterns of number of diagnosed mental conditions differ significantly between ADHD and non-ADHD individuals

**Figure 7.**
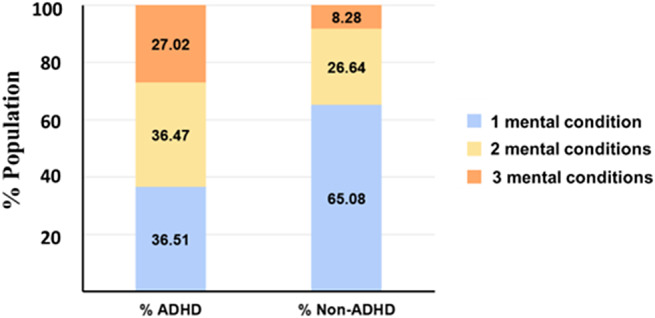
The figure presents a comparative analysis of the distribution of ADHD and non-ADHD individuals across three unspecified categories, represented by different colored segments (one mental condition – light blue, two mental conditions - yellow, three mental conditions – orange) within stacked bars.

To understand if the existence of multiple mental conditions is contingent on ADHD diagnosis, the number of mental conditions reported (1, 2, or 3) was compared between ADHD-diagnosed and non-ADHD populations. The distribution pattern is represented by different colored segments within stacked bars in Figure 7. For individuals diagnosed with ADHD, 36.51% fall into the category of only one mental condition, which is ADHD reported alone. Among ADHD patients, 36.47% reported the presence of an additional mental condition, while 27.02% reported the presence of an additional two mental conditions. For non-ADHD individuals, the distribution shifts significantly, with 65.08% reporting only one mental condition, 26.64% reporting two, and only 8.28% reporting a total of three mental conditions. This visual comparison highlights distinct differences in the distribution patterns between ADHD and non-ADHD populations across these categories, suggesting potential variations in demographic or behavioral characteristics associated with ADHD status.

### ADHD comorbidity exists with a significant positive association with ODD, PDD/ASD, and conduct disorder

**Figure 8.**
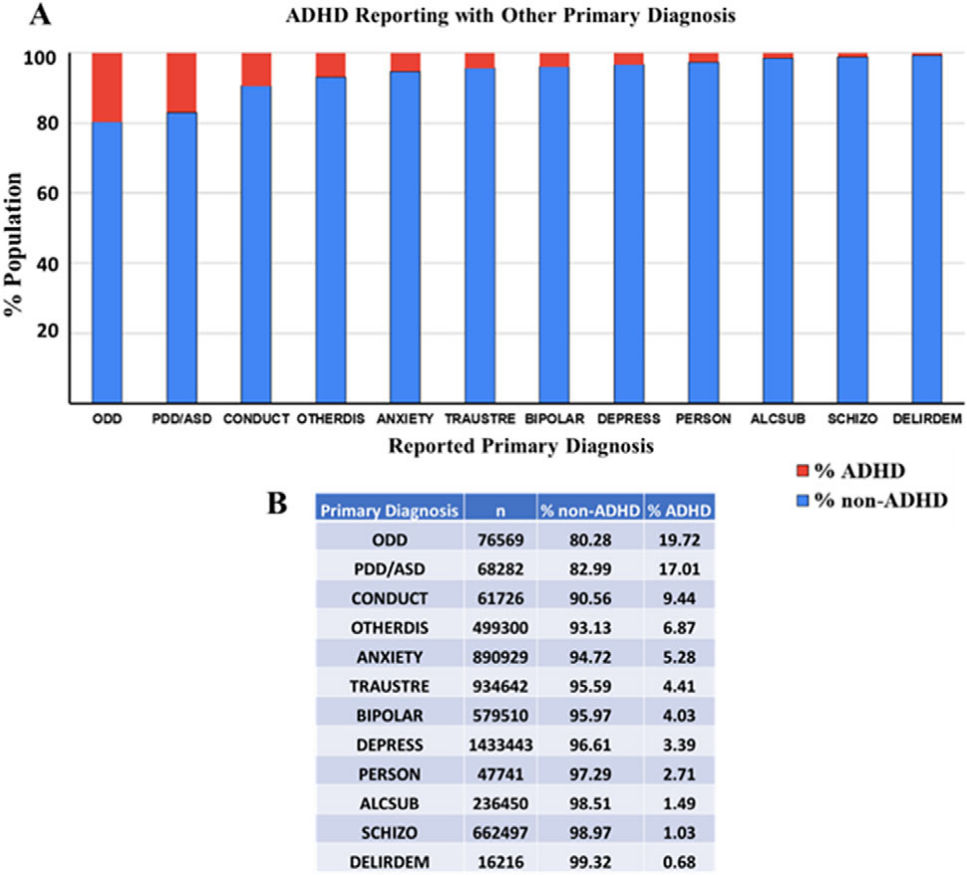
The bar chart presents a detailed visual representation of the prevalence of ADHD as % population within various primary diagnoses such as ODD, pervasive developmental disorder/autism spectrum disorder (PDD/ASD), conduct disorder, other disorders, anxiety disorders, trauma/stress-related disorders, bipolar disorder, depression, personality disorders, alcohol/substance use disorders, schizophrenia, and delirium/dementia in a given population in the year 2022. The population diagnosed with ADHD is represented in red, and those without ADHD are represented in blue. *Indicates a significant contingency between the primary diagnosis and ADHD (chi-square contingency test, *p* < 0.0001). The table provides the number of individuals (*n*) within each primary diagnosis and the corresponding percentages of non-ADHD and ADHD patients.

To assess ADHD comorbidity across different primary diagnoses ODD, pervasive developmental disorder/autism spectrum disorder (PDD/ASD), conduct disorder, other disorders, anxiety disorders, trauma/stress-related disorders, bipolar disorder, depression, personality disorders, alcohol/substance use disorders, schizophrenia, and delirium/dementia, the relative % population of ADHD diagnosis among the various primary diagnosis population was compared. Figure 8 clearly demonstrates that while ADHD comorbidity exists across various primary diagnoses, its prevalence is more pronounced in ODD, PDD/ASD and conduct disorder. ODD shows the highest ADHD prevalence at 19.72% among 76,569 individuals, indicating a strong association between these conditions. PDD/ASD also has a high ADHD prevalence at 17.01% among 68,282 individuals. Conduct disorder follows with a 9.44% ADHD prevalence among 61,726 individuals. For other disorders, the presence of ADHD is considerably lower, indicating that ADHD is more likely to co-occur with certain behavioral and developmental disorders. Thus, these findings suggest that ADHD is more commonly reported in conjunction with certain primary diagnoses, particularly ODD and PDD/ASD, while being less prevalent in conditions like schizophrenia and delirium/dementia. This highlights the importance of considering co-occurring conditions when diagnosing and treating ADHD, as the presence of certain primary diagnoses can significantly influence ADHD prevalence rates.

**Figure 9.**
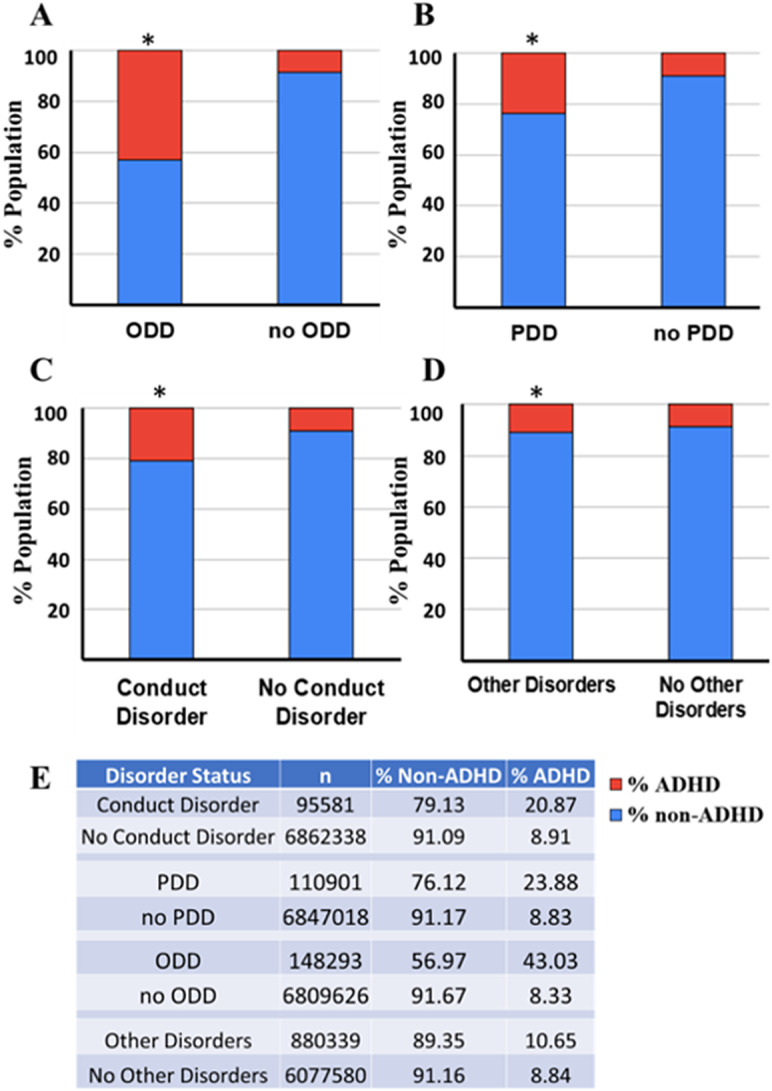
The figure effectively highlights the varying degrees of ADHD comorbidity across different primary diagnoses: ODD, PDD/ASD, conduct disorder, and other mental conditions. The population diagnosed with ADHD is represented in red, and those without ADHD are represented in blue. *Indicates a significant contingency between the primary diagnosis and ADHD (chi-square contingency test, *p* < 0.0001). The tables provide the number of individuals (*n*) within each diagnosis analyzed and the corresponding percentages of non-ADHD and ADHD patients.

To understand if higher ADHD prevalence among ODD, PDD, conduct disorder and other mental conditions is merely a chance or is contingent on the occurrence of this diagnosis, a further detailed analysis using the Pearson chi-square contingency test and odds ratio was performed. While the chi-square test helps analyze a significant association, the odds ratio provides the odds of one parameter being present along with the rate of occurrence when another parameter is present. The prevalence of % ADHD in the population with and without the diagnosis (ODD, PDD, conduct disorder and other mental conditions) was compared with each other. For ODD, the data shows that 43.03% of individuals with ODD (148,293 individuals) have ADHD, whereas only 8.33% of individuals without ODD (6,809,626 individuals) have ADHD (Figure 9a,e). The chi-square contingency test confirms that ODD and ADHD are contingent on each other (Pearson chi-square test, *p* < 0.0001). Further analysis of odds ratio calculations suggests that the odds of the ADHD diagnosis are 8.3 times higher if the patient is diagnosed with ODD.

Similarly, PDD has a high ADHD prevalence of 23.88% among 110,901 individuals, compared to 8.83% in those without PDD (6,847,018 individuals), highlighting a significant link between PDD and ADHD (Figure 9b,e). The chi-square contingency test confirms that PDD and ADHD are contingent (Pearson chi-square test, *p* < 0.0001), and the odds ratio calculations suggest that the odds of the ADHD diagnosis are 3.2 times higher if the patient is diagnosed with PDD. Conduct disorder also shows a high ADHD prevalence of 20.87% among 95,581 individuals, whereas the prevalence drops to 8.91% in those without conduct disorder (6,862,338 individuals) (Figure 9c,e). The chi-square contingency test confirms that conduct disorder and ADHD are contingent (Pearson chi-square test, *p* < 0.0001), and the odds ratio calculations suggest that the odds of the ADHD diagnosis are 2.7 times higher if the patient is diagnosed with conduct disorder. For other mental disorders, the reported category also shows a high ADHD prevalence of 10.65% among 880,339 individuals, whereas the prevalence drops to 8.84% in those without conduct disorder (6,077,580 individuals) (Figure 9d,e). The chi-square contingency test confirms that other mental disorders and ADHD are contingent (Pearson chi-square test, *p* < 0.0001), and the Odds ratio calculations suggest that the odds of the ADHD diagnosis are 1.2 times higher if the patient is diagnosed with conduct disorder.

These results underscore the heightened prevalence of ADHD in patients with ODD, PDD and conduct disorder, suggesting that these co-occurring conditions significantly increase the likelihood of an ADHD diagnosis. No other diagnosis was found significantly contingent on a positive association with ADHD diagnosis.

## Discussion

The analysis of the SAMHSA’s Mental Health Client-Level Data for the year 2022 provides critical insights into the prevalence and demographic distribution of ADHD among patients, revealing substantial implications for real-world applications and delving into the underlying reasons behind these findings. Out of 5,899,698 patients, 631,175 individuals were diagnosed with ADHD, representing 10.70% of the total patient population. This significant prevalence underscores the high demand for resources and support systems dedicated to ADHD diagnosis and treatment. The widespread nature of ADHD within the patient population emphasizes the need for comprehensive public health policies that address ADHD management effectively.

ADHD reporting shows a clear age-dependent decline, with the highest prevalence in the 0–11 age group (26.04%) and a gradual decrease across older age groups. This trend aligns with educational attainment, where ADHD reporting is highest in individuals with lower education levels and those in special education. The inverse relationship between ADHD prevalence and education level suggests that early intervention and continuous support throughout schooling can significantly impact the management of ADHD. This data highlights the importance of age-appropriate and education-level-specific interventions to support individuals with ADHD. The real-world implication here is the need for schools to implement robust support systems for students with ADHD, potentially including individualized education plans (IEPs), behavioral interventions and teacher training programs to recognize and support ADHD students effectively.

The analysis also shows that individuals with ADHD face significant challenges in employment. The highest prevalence of ADHD (6.71%) is among those not currently in the labor force. In contrast, full-time and part-time employees show significantly lower ADHD prevalence. This disparity underscores the need for workplace accommodations and employment support programs for individuals with ADHD. Addressing these employment challenges can help improve the overall quality of life and economic stability for individuals with ADHD. Employers should consider implementing flexible work schedules, providing clear and structured tasks and offering support services such as coaching or mentoring to help employees with ADHD thrive in the workplace.

The data analysis also suggests that ADHD prevalence varies significantly across racial groups, with the highest prevalence among Black individuals (9.71%) and the lowest among Asian individuals (5.05%). These variations indicate potential differences in genetic, environmental or sociocultural factors influencing ADHD diagnosis and management. Understanding these racial differences is crucial for developing culturally sensitive diagnostic criteria and treatment plans. For instance, healthcare providers might need to consider cultural attitudes towards mental health, potential biases in diagnosis and access to care when developing interventions for diverse populations. Public health campaigns tailored to specific communities can also help raise awareness and reduce the stigma associated with ADHD.

Geographically, the Midwest (10.43%) and the South (9.76%) show the highest ADHD prevalence, while the West (8.01%) and Northeast (8.04%) report lower rates. These regional differences suggest that local environmental factors, healthcare accessibility and socioeconomic conditions may influence ADHD prevalence. Regional strategies tailored to these specific conditions can enhance ADHD management and resource allocation. For example, areas with higher prevalence might benefit from increased funding for mental health services, more specialized training for healthcare providers and community-based support programs to address the specific needs of the population.

Additional demographic analysis indicates that males have a significantly higher ADHD prevalence (12.41%) compared to females (6.18%). This gender disparity necessitates gender-specific approaches in ADHD diagnosis and treatment. Additionally, marital status appears to influence ADHD prevalence, with the highest rates among never-married individuals (13.50%) and the lowest among married individuals (2.76%). These findings suggest that social support structures associated with marriage might play a protective role against ADHD symptoms. Interventions that enhance social support networks, such as group therapy or family counseling, could be particularly beneficial for individuals with ADHD. Moreover, living arrangements also impact ADHD prevalence, with individuals in private residences showing the highest rates (10.48%), compared to those experiencing homelessness (2.14%). This highlights the role of stable living conditions in managing ADHD and the need for supportive housing policies for those affected. Ensuring stable housing and providing resources for individuals transitioning out of homelessness can significantly improve their ability to manage ADHD and access necessary treatments.

The data reveals a significant positive association between ADHD and certain primary diagnoses, particularly ODD, PDD/ASD, conduct disorder and category of other mental conditions. Individuals with ODD are 8.3 times more likely to have ADHD, while those with PDD/ASD and conduct disorder are 3.2 and 2.7 times more likely, respectively. These findings emphasize the importance of comprehensive diagnostic assessments that consider potential comorbidities, as these co-occurring conditions can significantly impact the management and prognosis of ADHD. In clinical practice, this means that healthcare providers should be vigilant in screening for and addressing comorbid conditions, ensuring a holistic approach to treatment that addresses all aspects of the patient’s mental health.

The insights from this study underscore the critical need for tailored, multi-faceted approaches to ADHD management that consider demographic variables, comorbid conditions and socioeconomic factors. Healthcare providers should adopt comprehensive diagnostic and treatment protocols that address the diverse needs of individuals with ADHD. Policymakers should focus on enhancing healthcare accessibility, providing workplace accommodations and supporting educational interventions to improve outcomes for individuals with ADHD. This detailed analysis provides a robust foundation for targeted healthcare interventions and policy formulations, ultimately aiming to improve the management and quality of life for individuals with ADHD. In conclusion, the findings highlight significant demographic, socioeconomic and comorbidity-related variations in ADHD prevalence, underscoring the necessity for customized strategies to address these disparities. Effective management of ADHD requires a collaborative effort from educators, employers, healthcare providers and policymakers to create an inclusive and supportive environment for individuals with ADHD, enabling them to achieve their full potential.

## Conclusion

This study’s analysis of the 2022 data from SAMHSA’s Mental Health Client-Level Data reveals the extensive prevalence of ADHD, affecting 10.70% of the patient population. The findings underscore a significant demand for dedicated ADHD resources and support systems. The age-dependent decline in ADHD prevalence, highest among children aged 0–11 and gradually decreasing with age, indicates the need for early intervention and continuous support through educational systems. Schools should implement individualized education plans and behavioral interventions, supported by trained teachers, to manage ADHD effectively from a young age.

Employment status significantly impacts ADHD prevalence, with the highest rates among individuals not currently in the labor force, highlighting the challenges in securing and maintaining employment for those with ADHD. Employers can help by implementing flexible work schedules, providing clear and structured tasks and offering support services such as coaching. Additionally, racial disparities in ADHD prevalence point to potential differences in genetic, environmental or sociocultural factors, with Black individuals showing the highest rates and Asian individuals the lowest. These variations necessitate culturally sensitive diagnostic criteria and treatment plans, alongside tailored public health campaigns to raise awareness and reduce stigma. Geographically, ADHD prevalence varies, with the Midwest and South reporting the highest rates, suggesting that local environmental factors and healthcare accessibility play significant roles. Gender disparities, with males showing higher prevalence, and the influence of marital status, where never-married individuals have the highest rates, further emphasize the need for personalized interventions. Living conditions also impact ADHD prevalence, with higher rates in private residences compared to homelessness.

The study also highlights a strong association between ADHD and comorbid conditions such as ODD, PDD/ASD and conduct disorder, underscoring the necessity for comprehensive diagnostic assessments considering potential comorbidities. In conclusion, the findings highlight significant demographic, socioeconomic and comorbidity-related variations in ADHD prevalence. Effective management of ADHD requires a collaborative effort from educators, employers, healthcare providers and policymakers to create an inclusive and supportive environment for individuals with ADHD. Tailored, multifaceted approaches considering demographic variables, comorbid conditions and socioeconomic factors are essential to address these disparities and improve the quality of life for individuals with ADHD.

## Data Availability

All data reported in this paper are publicly available at: https://www.samhsa.gov/data/data-we-collect/mh-cld-mental-health-client-level-data.
