# Supplementary material for: Assessing ADHD prevalence and comorbidities in the United States: Insights from the Substance Abuse and Mental Health Services (SAMHSA) data
Source: Glob Ment Health (Camb). 2024 Dec 6;11:e121. doi: 10.1017/gmh.2024.104 (PMC11704367; doi:10.1017/gmh.2024.104)
Supplement: Peer review history

# Editor report: Recommendation: Assessing ADHD prevalence and comorbidities in the United States: Insights from the Substance Abuse and Mental Health Services (SAMHSA) data — R0/PR2

**DOI:** 10.1017/gmh.2024.104.pr2

**Affiliations:** Psychiatry, Makerere University CHS, Uganda

Dear Authors,

Please respond to the very minor comments from reviewer 1

# Editor report: Decision: Assessing ADHD prevalence and comorbidities in the United States: Insights from the Substance Abuse and Mental Health Services (SAMHSA) data — R0/PR3

**DOI:** 10.1017/gmh.2024.104.pr3

**Affiliations:** Johns Hopkins University Bloomberg School of Public Health, United States

No accompanying comment.
